# Atraumatic Cervical Disc Herniation With Rapidly Progressive Myelopathy in a 47-Year-Old Male: A Case Report

**DOI:** 10.7759/cureus.74152

**Published:** 2024-11-21

**Authors:** Brian A Parker, Cody A Cunningham, Abhijith R Bathini, Naresh P Patel, Wayne A Martini

**Affiliations:** 1 Internal Medicine, Mayo Clinic Arizona, Phoenix, USA; 2 Neurosurgery, Mayo Clinic Arizona, Phoenix, USA; 3 Emergency Medicine, Mayo Clinic Arizona, Phoenix, USA

**Keywords:** case report, cervical spinal stenosis, cord compression, disc herniation, myelopathy, radiculopathy, spinal surgery

## Abstract

Atraumatic acute myelopathy caused by idiopathic disc herniation is rare. This case presents a 47-year-old male with a sudden onset of severe neck pain and weakness upon waking that progressively worsened. His rapidly progressive myelopathy led to an MRI of the cervical spine, revealing severe spinal canal stenosis at the C6-C7 level due to a large disc herniation deforming the spinal cord. The patient underwent anterior cervical discectomy and fusion (ACDF) at the C6-C7 level. Postoperatively, he showed significant improvement in pain and paresthesia, though some residual numbness and balance issues persisted.

This case highlights the rapid progression and severe neurological impact of cervical spinal stenosis due to disc herniation. Despite reassuring findings on initial CT imaging, the patient’s rapidly worsening symptoms on reassessment prompted an MRI, which confirmed the diagnosis and highlighted the urgent need for surgical intervention. The successful outcome for this patient is largely due to the rapid identification and surgical decompression of his severe cervical spinal cord compression.

Cervical spinal stenosis, particularly when associated with disc herniation, can lead to profound, irreversible neurological impairment if not promptly addressed. This case demonstrates the critical need for early clinician identification and surgical intervention to prevent permanent deficits and improve patient outcomes.

## Introduction

Cervical spinal stenosis is a condition characterized by the narrowing of the spinal canal in the cervical region, which can lead to compression of the spinal cord and nerve roots. It is commonly associated with degenerative changes in the spine, such as disc herniation, osteophyte formation, and ligamentous hypertrophy. While cervical spinal stenosis typically presents with neck pain and radicular symptoms, severe cases can progress to myelopathy, a condition that can cause significant neurological deficits. Although most cases of disc herniation causing myelopathy occur in the lumbar or thoracic regions, cervical disc herniation can also lead to acute myelopathy, albeit less commonly [1].

Cervical radiculopathy, often a symptom of cervical spinal stenosis, is a common condition, affecting 85 out of 100,000 people annually [1]. While degenerative changes are common with aging, they don't always cause symptoms [1,2]. The frequency of myelopathy, however, does increase with age, with a more pronounced increase in individuals over 70 years old. The location of myelopathy also shifts upward with age, becoming more common at the C3-C4 level in older individuals, unlike younger patients where C5-C6 is more frequently affected [2].

## Case presentation

A 47-year-old Caucasian male with a past medical history of hypertension, hyperlipidemia, and type 2 diabetes mellitus presented to the emergency department (ED) with complaints of severe neck pain radiating down his entire body. He reported the onset of paresthesia in both hands and legs that started on the last day, along with back pain that had progressively worsened over the past few weeks. The patient specifically noted an inability to flex his proximal muscles bilaterally due to pain, with an associated shocking sensation running down his neck to his toes when extending his feet. He reported a sensation of pins and needles in the lateral aspect of both upper extremities, though he felt like his strength was intact. In the morning prior to coming to the ED, the patient woke up unable to bear weight on his legs, resulting in a ground-level fall. He required assistance from his family to lift him and be transported to our ED.

On physical examination, the patient appeared in distress, particularly when attempts were made to flex or extend his legs. His upper extremities were found to have full range of motion at the wrist and elbow; however, abduction of the shoulders created a similar shocking sensation to the one he felt with the movement of his legs. Neurological examination revealed diminished sensation in both hands and feet, with no significant muscle weakness. Reflexes were slightly brisk in the lower extremities, raising concern for a potential upper motor neuron lesion. He had significant difficulty standing with a wide, bowed stance and shaking due to his lower extremity muscle weakness.

Given the patient's symptoms and examination findings, imaging studies were promptly ordered. A CT scan of the cervical spine without contrast showed multilevel degenerative changes, without evidence of acute fractures or subluxation. There was mild bony neural foraminal stenosis on the right at C6-C7 but no significant central canal stenosis. However, given the severity of his symptoms as well as their progression, an MRI of the cervical spine and thoracic spine with and without contrast was ordered and 10 milligrams (mg) of dexamethasone was administered intravenously (IV). The differential diagnosis for this patient initially included various causes of cervical radiculopathy and myelopathy, such as cervical spondylosis, disc herniation, spinal cord tumor, and inflammatory or infectious processes.

**Figure 1:**
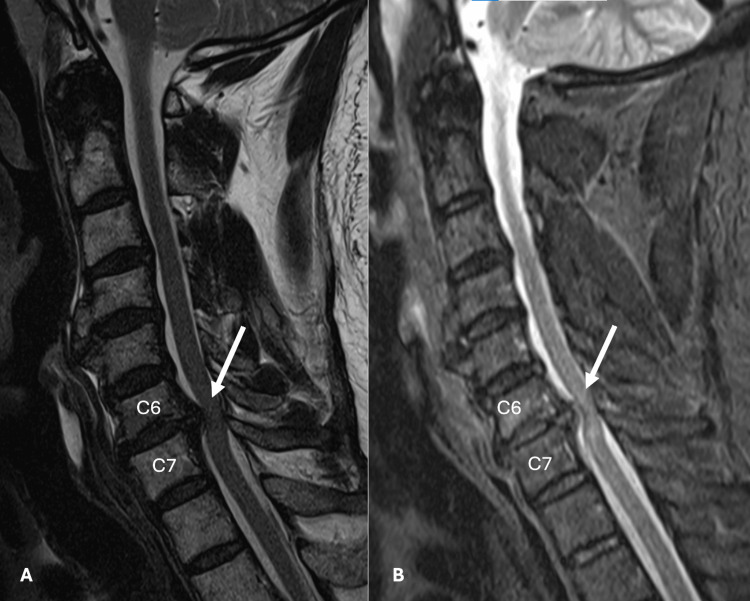
Sagittal T2 (A), T2 STIR (B) sequences showing a large posterior disc herniation at C6-7 resulting in severe canal stenosis with compression of the spinal cord.

**Figure 2:**
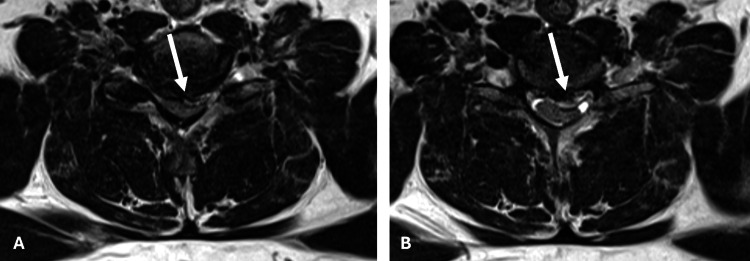
Axial T2 sequences showing a large disc herniation at C6-7, eccentric to the left, resulting in severe canal stenosis and spinal cord compression.​ Pane A: above the area of disc herniation. Pane B: at the level of disc herniation.

MRI of the cervical spine revealed severe central canal stenosis at the C6-C7 level due to a large disc herniation deforming the spinal cord (Figures 1, 2). The patient was evaluated by neurosurgery and given the severe spinal cord compression that correlated to the patient’s symptoms, surgical intervention was recommended. Over the next 8-12 hours, the patient began to experience worsening weakness in his lower extremities and urinary retention with post-void residual > 500 mL in the bladder. The patient underwent a C6-7 anterior cervical discectomy and fusion (ACDF) for spinal cord decompression.

Intraoperatively, the patient was noted to have a large herniated disc associated with a piece of cartilaginous endplate from the vertebral body compressing the ventral aspect of the spinal cord. After the disc was extracted, the posterior longitudinal ligament (PLL) was opened to allow for wide decompression all the way to the neuroforamina bilaterally. An interbody spacer was placed followed by cervical plate fixation to facilitate the fusion process.

The surgery was highly successful in decompressing the spinal canal and stabilizing the affected segment. Postoperatively, the patient reported a significant reduction in pain and paresthesia, though he continued to experience some residual numbness in his hands and feet. On post-operative day 5, sensation in his lower extremities was still altered and he had some trouble with balance; however, he was able to ambulate 100 feet with a walker and was demonstrating increased upper extremity weight-bearing and improved gait speed and cadence.

**Figure 3:**
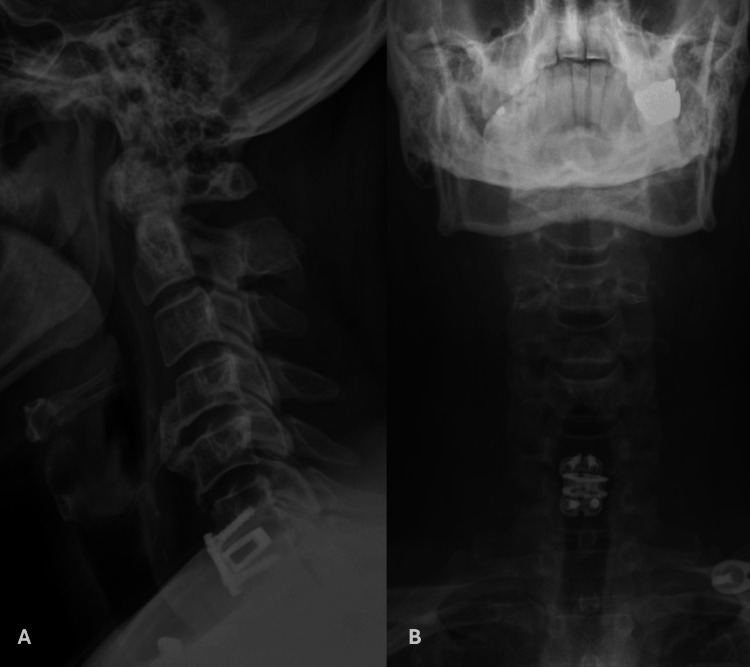
Postoperative X-rays showing appropriate alignment of the interbody spacer along with cervical plate and screws​ Pane A: Lateral cervical spine X-ray. Pane B: Anterior-posterior cervical spine X-ray.

He was transferred to an inpatient rehab with a cervical collar for stabilization and instructed to avoid strenuous activities. Postoperative X-rays showed a stable construct with maintained alignment and appropriate placement of the cervical instrumentation (Figure 3).

## Discussion

Non-traumatic cervical myelopathy with rapid progression towards paralysis, as seen in this case, can often prove challenging to diagnose and treat because of its rare occurrence, lack of a clear inciting incident, and difficulty of establishing the diagnosis without MRI imaging. This case highlights the importance of prompt recognition and intervention in patients who present with symptoms of cervical radiculopathy and myelopathy, which, if left untreated, will likely result in catastrophic neurological deficits. 

Only 12 reported cases of atraumatic cervical myelopathy exist in the literature, and only 4 involve C6-7 [3-6]. The unique aspect of this case in particular is the rapid clinical progression from neck pain and paresthesia to profound lower extremity weakness, bladder dysfunction, and ultimately paraplegia within the first 12 hours from symptom onset. As a result, it is the only case where the time of symptom onset to surgical intervention was less than 48 hours. All other studies have time ranges from 2 weeks to 7 months. 

This case highlights the importance of clinical diagnosis rather than relying on imaging to diagnose myelopathy. CT imaging was largely unremarkable and discharge from the ED instead of admission would have proven devastating for the patient. Furthermore, there are many hospital systems that are resource-poor, where MRI imaging may take days or weeks to schedule if the diagnosis is not firmly made from a clinical standpoint; such a delay in this patient would likely cause progression to total paralysis as documented in the case by Suzuki et al [4]. 

For clinicians, this case highlights the importance of clinical assessment over imaging alone. Although initial CT findings were unremarkable, reliance on clinical judgment prompted urgent MRI and neurosurgical consultation, which proved critical for the patient's outcome. In resource-limited settings where MRI may not be readily available, clinical suspicion and early decision-making are vital, as delays in imaging can lead to devastating outcomes, such as total paralysis. Clinicians must perform a thorough exam and prioritize patients with neurological symptoms consistent with myelopathy for expedited imaging and intervention.

In our case, a preoperative MRI revealed multilevel degenerative changes and spinal cord compression at C6-C7, which aligns with findings in Lourie et al.’s discussion on central cervical soft disc herniation syndrome. These findings, in conjunction with the patient's neurological decline, suggest that the spinal cord was not only mechanically compressed but may have also started suffering ischemic damage due to impaired blood flow to the anterior spinal artery and/or its branches [5]. 

The outcome of this case reinforces the importance of urgent surgical intervention, as highlighted in the cases which ultimately resulted in neurologic demise [3,6,7]. Early decompression is crucial to avoid the irreversible neurologic damage that may result from prolonged compression and ischemia. In our case, timely anterior cervical decompression and fusion at C6-C7 successfully relieved the spinal cord compression, and although residual sensory deficits persisted postoperatively, the patient fully regained all bladder and muscular function. 

In conclusion, this case adds to the growing body of literature on nontraumatic cervical myelopathy and underscores the critical importance of early diagnosis and intervention. The onset of symptoms described by patients may often be mild and include benign descriptions such as neck pain or some weakness. Furthermore, there may be no clear precipitating activity that triggers the event. The constellation of symptoms comprising gait instability, tingling, weakness, or reflex abnormalities in the setting of neck pain should always raise suspicion for potential cervical myelopathy. Clinicians must perform a thorough clinical assessment to determine if admission and/or MRI imaging is warranted, as timely decompression can prevent permanent neurological deterioration. 

## Conclusions

Cases of atraumatic cervical disc herniation leading to myelopathy are uncommon and present unique diagnostic and management challenges for clinicians. This case highlights the vital role of a thorough physical examination and clinical judgment in suspecting cervical myelopathy even when imaging results are inconclusive. In many settings, especially those with limited resources, MRI may not be immediately accessible, emphasizing the necessity for clinicians to rely on clinical assessment to prioritize urgent diagnostic and surgical interventions. In this patient's case, rapid intervention through anterior cervical discectomy and fusion at the C6-C7 level enabled substantial recovery, though some sensory deficits and balance issues persisted.

By documenting this rare and rapidly progressing case of atraumatic cervical myelopathy, this report contributes to the growing literature and emphasizes the importance of clinician awareness of cervical disc herniation as a potential cause of acute myelopathy. Clinicians must perform a thorough neurological exam, and maintain a high index of suspicion in patients presenting with non-specific symptoms. Neck pain, gait instability, or sensory abnormalities may be early indicators of significant spinal pathology. Early identification and intervention are paramount in preventing permanent neurological impairment and optimizing patient outcomes, as illustrated by the trajectory of the patient’s recovery post-surgery.
